# The BCR-ABL/NF-κB signal transduction network: a long lasting relationship in Philadelphia positive Leukemias

**DOI:** 10.18632/oncotarget.11507

**Published:** 2016-08-22

**Authors:** Giovanna Carrà, Davide Torti, Sabrina Crivellaro, Cristina Panuzzo, Riccardo Taulli, Daniela Cilloni, Angelo Guerrasio, Giuseppe Saglio, Alessandro Morotti

**Affiliations:** ^1^ Department of Clinical and Biological Sciences, University of Turin, Orbassano, Italy; ^2^ Department of Oncology, University of Turin, Orbassano, Italy

**Keywords:** BCR-ABL, NF-κB, IκB-α, NFKBIA, CML

## Abstract

The Nuclear Factor-kappa B (NF-κB) family of transcription factors plays a key role in cancer pathogenesis due to the ability to promote cellular proliferation and survival, to induce resistance to chemotherapy and to mediate invasion and metastasis. NF-κB is recruited through different mechanisms involving either canonical (RelA/p50) or non-canonical pathways (RelB/p50 or RelB/p52), which transduce the signals originated from growth-factors, cytokines, oncogenic stress and DNA damage, bacterial and viral products or other stimuli. The pharmacological inhibition of the NF-κB pathway has clearly been associated with significant clinical activity in different cancers. Almost 20 years ago, NF-κB was described as an essential modulator of BCR-ABL signaling in Chronic Myeloid Leukemia and Philadelphia-positive Acute Lymphoblastic Leukemia. This review summarizes the role of NF-κB in BCR-ABL-mediated leukemogenesis and provides new insights on the long lasting BCR-ABL/NF-κB connection.

## INTRODUCTION

The Philadelphia chromosome (Ph^+^) refers to the translocation t(9;22) and is the genetic hallmark of chronic myeloid leukemia (CML) and of about one third of acute lymphoblastic leukemias (ALL) [1-5]. Ph^+^ chromosome codes for the BCR-ABL chimeric protein which is characterized by the constitutive activation of the tyrosine kinase ABL [6-9]. The generation of the p210-BCR-ABL isoform is responsible for the CML phenotype, while the shorter p190-BCR-ABL isoform leads to the development of Ph^+^ ALL [10]. However, the presence of p210-BCR-ABL isoform is not restricted to CML, as it is found in 10% to 20% of adults and in a small percentage of children with ALL[11-13]. Although BCR-ABL tyrosine kinase inhibitors (TKIs) have undoubtedly revolutionized the therapy of Ph^+^ leukemias [14,15], both CML and ALL are not completely eradicated by BCR-ABL inhibition, as extensively reviewed elsewhere [16-20]. In particular, the major unmet clinical needs in the field of Ph^+^ leukemias are represented by: i) the incomplete eradication of CML chronic phase, ii) the need for a better control of CML blast phase [21] and iii) the improvement of TKIs efficacy in Ph^+^ ALL [22]. Therefore, the molecular dissection of BCR-ABL signaling network is advisable to identify the signaling axes which are essential for the maintenance of Ph^+^ leukemias and whose inhibition allows to achieve synthetic lethality together with BCR-ABL TKIs. Synthetic lethal therapies, designed to target those synthetic lethal partners of genetic aberrations found in cancers, allow to selectively target cancer cells while sparing normal cells, as extensively reviewed [23]. In accordance with this notion, tandem inhibition of NF-κB signaling proteins together with potential synergistic pathways has always appeared as an attractive strategy. The Nuclear Factor-kappaB (NF-κB) family of transcription factors was originally investigated in the immune system due to its ability to regulate the expression of cytokines and effector enzymes, especially in response to the activation of several receptors involved in immunity, including T and B-cell receptors [24, 25]. Furthermore, the NF-κB pathway has been linked to the regulation of several processes involved in tumorigenesis [26,27]. These include proliferative signaling [28], cell death evasion [29, 30] and resistance to chemotherapy [31]. Therefore NF-κB signaling was early identified as a potential target for cancer therapy [32,33]. Moreover, almost 20 years ago NF-kB was described as a key BCR-ABL partner [34-36]. Since then, various reports have further investigated the contribution of different NF-κB components in CML and Ph^+^ ALL pathogenesis. These results have allowed a better understanding of NF-κB signaling and have drawn particular attention on its targeting in Ph^+^ leukemia context. In this review, we will consider the BCR-ABL/NF-κB crosstalk with relation to the development and maintenance of leukemia and we will debate the potential therapeutic strategies to block NF-κB signaling in Ph^+^ malignancies.

### NF-kB signaling

**Figure 1:**
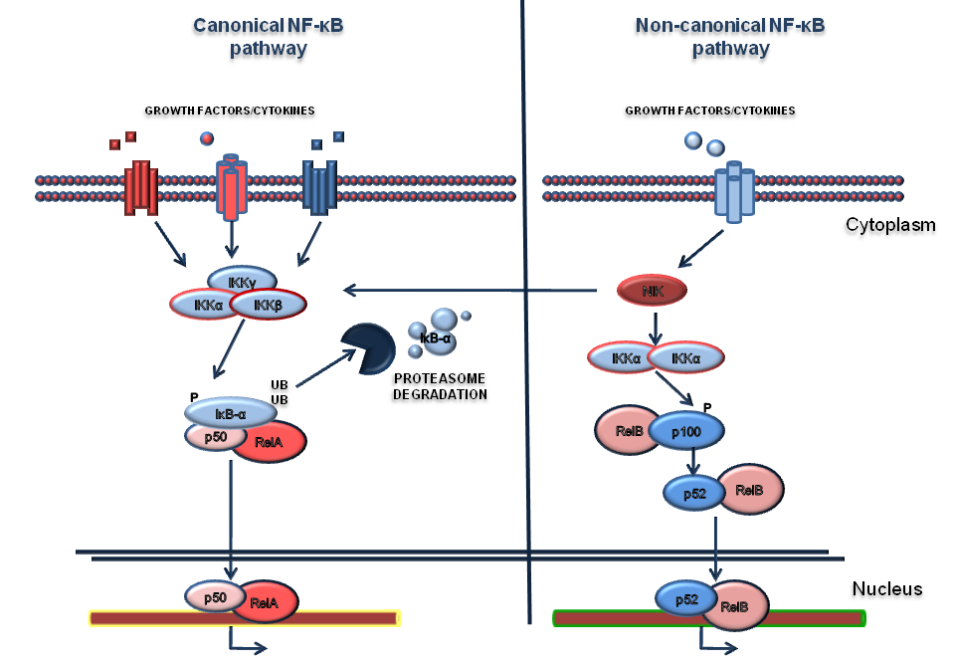
NF-κB pathway Canonical and non-canonical NF-κB pathways are represented in the *left* and in the *right* of the figure respectively. The activation of canonical pathway is mediated by various ligands such as Tumor Necrosis Factor (TNFα), Interleukin-1 (IL-1), or growth factors. The activation relies on the phosphorylation of IκB-α by the IKK complex and subsequent its degradation by the proteasome. Consequently, the RelA/p50 complex translocates to the nucleus where it activates the transcription of target genes. The non-canonical pathway is based on the activation of IKKα by the NF-κB-inducing kinase (NIK), after stimulation. In turn the complex NIK-IKKα phosphorylates the p100 subunit. As a consequence, p100 is processed in a proteasome dependent manner, generating the subunit p52. This event results in the activation of p52-RelB that induces the transcription of distinct target genes.

The Nuclear Factor-kappaB (NF-κB) family of transcription factors mediates various biological processes which can be deregulated in cancer pathogenesis and involved in generating resistance to chemotherapy. Consequently, the experimental dissection of the NF-κB signaling network offers new opportunities to exploit therapeutically the inhibition of the pathway [27, 37-41]. NF-κB members comprise two protein subfamilies which are synthesized as large precursors, namely Rel and NF-κB. In turn, the Rel family includes RelA (also known as p65), RelB and c-Rel, while NF-κB members are p50 (that originates from the p105 precursor) and p52 (that originates from the p100 precursor). They are also called NF-κB1 and NF-κB2 respectively and are able to form hetero- and homo-dimers. The NF-κB pathway is modulated by negative or positive regulatory elements canonical and non-canonical pathways (Figure 1). The canonical pathway is based on the negative regulation of IκB-α [42, 43], which retains p65/p50 heterodimers in the cytoplasm in a latent state, thus inhibiting their binding to DNA. Various stimulations such as cytokines, growth factors, LPS and other stimuli, promote the activation of the IKK kinase complex, which in turn phosphorylates IκB-α on serine residues [44-46]. This event primes IκB-α to ubiquitination and its consequent degradation through the proteasome. Upon IκB-α degradation, p65/p50 complex translocates into the nucleus where it binds to the enhancer or promoter regions of specific DNA sequences and promotes transcription of target genes. NFKBIA, the gene encoding for IκB-α, is one of the first genes to be induced, providing a mechanism of negative feedback [47, 48]. The non-canonical pathway relies on the p50 and p52 subunits, as reviewed elsewhere [49,50]. In particular, the kinase NIK (NF-κB inducing Kinase) is able to activate IKK-α complex which mediates p100 phosphorylation. This results in the proteasome-dependent processing of p100 to p52, driving activation of RelB/p52 heterodimers that in turn target distinct κB elements on DNA[51]. NF-κB is known to regulate several cellular processes, including proliferation [28], protection from apoptosis [29, 30, 52], cell cycle [53], cell migration and angiogenesis [54] and resistance to chemotherapy[31]. Due to this broad spectrum of effects, deregulation of NF-κB signaling has been strongly associated with the development of several diseases, including hematological cancers [55-57]; however, NF-κB family members mutations, which induce its constitutive activation, are rare events in hematological malignances [55]. It is clear that the two pathways of NF-kB differ in their signaling mechanisms. Moreover, increasing evidence suggests that the non-canonical pathway regulates important biological functions in the lymphoid compartment, such as lymph node organogenesis, B-cell development and survival and it is also found deregulated mostly in lymphoid malignancies [58]. In addition, it should be noted that the activation of NF-κB is in most cases a consequence of several other aberrantly activated pathways (for example EGF-R [59], HGF/c-MET [60], oncogenic Ras [61, 62], PI3K [63], AKT [64], SRC [65] and others).

## HISTORICAL PERSPECTIVE ON BCR-ABL/NF-κB RELATIONSHIP

### Cell lines and cellular models

**Figure 2:**
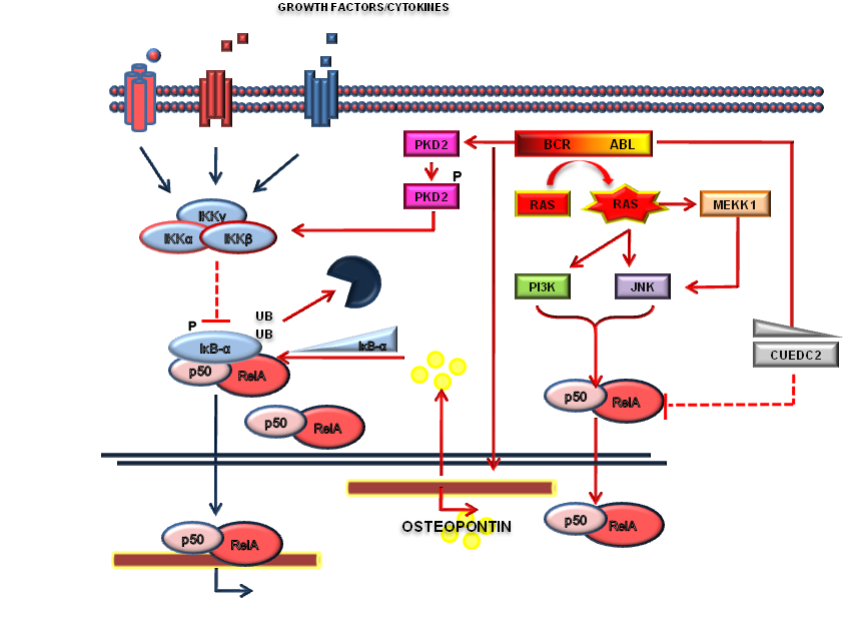
BCR-ABL/NF-κB crosstalk Numerous pathways, including Ras, MEKK1, c-Jun N-terminal kinase (JNK) and protein kinase D2 (PKD2) are activated by the Bcr-Abl and converge on NF-κB activation. NF-κB activation is also under the regulation of osteopontin or CUEDC2, as indicated.

The first observations that Bcr-Abl regulates the NF-κB signaling were made with the p210-Bcr-Abl-transformed DA1, an IL-3-dependent murine cell line [34]. In this cellular model, Bcr-Abl expression abrogated IL-3-dependent growth and enabled NF-κB to bind to DNA-responsive elements. Notably, the inhibition of RelA by antisense oligonuclotides reverted the IL-3-independence of Bcr-Abl-transformed DA1 cells, suggesting that NF-κB may contribute to Bcr-Abl-mediated tumorigenesis. Similar conclusions were drawn using the Bcr-Abl-expressing 32D cell line [35], an Il-3 dependent murine myeloblast-like cell line, where Reuther and colleagues further dissected the mechanisms of NF-κB activation by Bcr-Abl showing that Ras is required for the regulation of NF-κB. In particular, RasA17 mutant, acting as a dominant negative protein, affects the ability of BCR-ABL to activate NF-κB. Notably, expression of an IκB-α isoform unable to be degraded, prevented NF-κB activation by Bcr-Abl. Thus, in the specific context of Bcr-Abl-dependent cells, NF-κB appeared unnecessary to protect cells from death caused by IL-3 removal or treatment with DNA damage agents. However, a murine xenotransplantation model of Bcr-Abl-32D cells showed that NF-κB activation was necessary to mediate Bcr-Abl tumorigenesis, suggesting that *in vitro* cellular models may harbor mechanistic bias for the study of NF-κB signaling [35]. Other groups have subsequently confirmed the Bcr-Abl/NF-κB connection [66] *in vitro*. Using a Bcr-Abl-BaF3 cell line, a Il-3 dependent murine pro B cell line, it was shown that NF-κB activation is partially dependent by IκB-α degradation but independent of IκB kinase (IKK) activity [36], while Ras appeared necessary for the activation of NF-κB. This work described for the first time NF-κB activation in primary CML blast crisis [36]. In addition to the essential role of Ras, it was also demonstrated that various and complex mechanisms were responsible for triggering NF-κB activity. In particular, NF-κB activation is also dependent on downstream targets of Bcr-Abl including MEK kinase-1 (MEKK1). Bcr-Abl enhances MEKK1 expression and kinase activity which in turn strongly induces NF-κB signaling. On the contrary, inhibition of MEKK1 with a dominant-negative mutant decreased NF-κB activation [67]. Bcr-Abl can induce activation of the NF-κB pathway in Bcr-Abl cells by Protein Kinase D2 (PKD2). It has been established that Bcr-Abl induces activation of NF-κB in LAMA84 cells, a human CML blast crisis cell line, through tyrosine phosphorylation of PKD2 [68] at Tyr438. Among the mechanisms by which NF-κB can be activated by Bcr-Abl it is worth mentioning that osteopontin -a protein produced and secreted by Bcr-Abl-expressing cells- can bind integrin receptors and regulate IκB-α expression in an autocrine manner [69]. Finally, by analyzing NF-κB network activity, it was pointed out a negative association between the expression of CUEDC2, which is associated with endocrine resistance in breast cancer, and NF-κB signaling. In particular knockdown of CUEDC2 in K562 cell line, a CML blast crisis cell line, causes an increase of IKK complex phosphorylation and favors IκB-α degradation [70]. Altogether, these original contributions clearly suggest that: i) BCR-ABL is able to promote NF-κB activation *in vitro*; ii) Ras is necessary for NF-kB activation; iii) the contribution of IκB-α and IKK in the regulation of NF-κB is not entirely clear -at least in cell lines- and needs further studies in murine models (Figure 2).

### Primary Philadelphia positive samples

In human-derived primary Ph^+^ leukemias the assessment of NF-κB activation led to different conclusions. In an original report based on electrophoretic mobility shift assay (EMSA) analysis, NF-κB was found highly active in CML blast crisis [36]. Similarly, other analyses with a group of myeloid neoplasms, including AML, chronic phase CML and blast phase CML, described that NF-κB is markedly active in CML blast crisis when compared to the chronic phase of the disease[71]. However, successive works which adopted different experimental approaches to measure NF-κB activity, showed that even the chronic phase of CML is associated with an increased NF-κB activation [72] when compared to normal bone marrow samples, suggesting a functional heterogeneity. Conversely, in Ph^+^ ALL, constitutive NF-κB activation may be considered a common feature of the disease [73]. As a general principle, NF-κB is more active in Ph^+^ ALL and CML blast crisis with respect to the chronic phase disease. Apparent discrepancies among different reports could be explained either accordingly to different sensitivity of the methods used or related to population heterogeneity. Undoubtedly, the data available so far highlight the relevant role of NF-κB (regulation) in Ph^+^ leukemias.

### Murine models

Besides cell lines, murine models supply a good platform for studying the role of NF-κB in the pathogenesis and development of leukemias [74, 75]. Van Etten and colleagues showed in mouse models of CML and Ph^+^ B-ALL that NF-κB contributes to myeloid and lymphoid leukemogenesis through Bcr-Abl [76]. Primary bone marrow murine hematopoietic cells were infected to express both BCR-ABL and an IκB-α mutant (IκBαSR) that stabilizes NF-κB complex in the cytosol or dominant-negative, kinase-inactive, mutants of IKK [76]. Infections were performed to recapitulate Ph^+^ ALL and CML-like phenotypes. In these models, IκBαSR and IKK mutants were shown to attenuate Bcr-Abl-mediated leukemogenesis and to prolong survival. Notably, authors demonstrated that NF-kB inhibition affects BCR-ABL leukemogenesis partially through the impairment of leukemic stem cells. Specifically, IκBαSR and IKK mutants decrease the number of leukemia stem cells capable of initiating tumorigenesis [76]. Furthermore, using limiting dilution secondary transplantation of BM from CML mice, authors clearly demonstrated that SR-IkBα and IKKα mutant significantly affect the number of cells that are able to recapitulate the disease in the secondary recipient. Beside demonstrating that IkBα and IKK are essential components of the BCR-ABL leukemogenesis, this work also demonstrated that SRIkBα and IKK mutants significantly increase the sensitivity of Ph^+^ cells to TKI. This work provided the first evidence that *in vivo* Bcr-Abl activates NF-κB through the classical (canonical) IKK/IκB-α pathway and strongly supports the rationale that therapies designed to target canonical NF-κB network may be effective in Ph^+^ leukemias. Furthermore, this preclinical *in vivo* model rules out most of the controversial results obtained with cellular models, and offers the chance to design clinical trials with IKK inhibitors in combination with TKI in highly challenging settings, such as the therapy of Ph^+^ ALL and CML blast crisis.

### The contribution of the microenvironment

**Figure 3:**
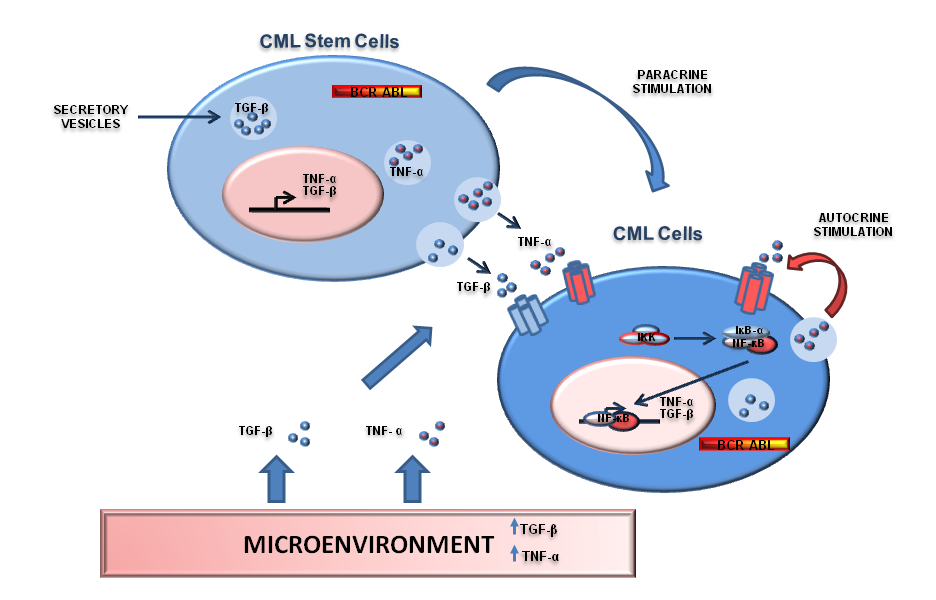
NF-κB is regulated by the environment where Ph+ positive cells reside Interactions between Ph^+^ stem/progenitor cells and stromal cells and representation of TGF-β and TNF-α networks. Both TGF-β and TNF-α can be secreted by CML cells as part of a autocrine/paracrine loop able to sustain NF-kB signaling. Moreover, Ph^+^ CML cells can be regulated by TGF-β and TNF-α produced by stromal cells or inflammatory cells. This mechanism may promote NF-kB signaling in a BCR-ABL-independent manner.

NF-κB signaling is also under the control of several cytokines produced by stromal cells or by the tumor itself [42]. The Tumor Necrosis Factor-α (TNF-α) is one of the most important cytokines able to activate NF-κB [77]. At the same time, TNF-α signaling was shown to be affected by Bcr-Abl [78]. In particular, ectopic expression of BCR-ABL promoted TNF-α receptor down-regulation with consequent impairment of TNF-α signaling. Recently, it was demonstrated that CML stem/progenitor cells secrete TNF-α in a Bcr-Abl Kinase-independent manner [79] promoting NF-κB activation. Specifically, autocrine TNF-α production is able to sustain CML stem cells and progenitor cells survival and pharmacological targeting of the TNF-α/NF-κB pathway is able to synergize with Bcr-Abl inhibition to a relevant degree. Again, these observations point to a key role of the microenvironment in the regulation of NF-κB signaling in CML [79]. Similarly to TNF-α, the TGF-β signaling was also shown to play an essential role in CML biology [80-82]. Notably, TGF-β orchestrates CML cellular destiny through a complex PI3K/AKT/NF-κB/MMP9 pathway, which includes NF-κB [83]. Overall, these data suggest that NF-κB is regulated by the microenvironment where Ph^+^ positive cells reside. In particular, both autocrine TNF-α secretion and cytokines produced from the environment are able to sustain NF-κB signaling in CML cells. The presence of a Bcr-Abl independent mechanism of NF-κB activation in Ph^+^ leukemias has two important implications. Firstly, it suggests that non-cell autonomous or micro-environmental processes are important in the maintenance of leukemias and therefore must be considered to design an effective targeted therapy for these cancers. Secondarily, the contribution of the stroma in the activation of NF-κB may also explain most of the controversial results observed in cell lines *vs* primary samples *vs* murine models. For instance, IKK and IκB- α appeared dispensable for NF-κB activation in some cell line models, while are clearly important in murine models or in primary cells. These discrepancies may be explained by the absence of the microenvironment, a source of TNF-α and TGF-β , in cell line experiments (Figure 3).

## THERAPEUTICAL IMPLICATIONS

**Figure 4:**
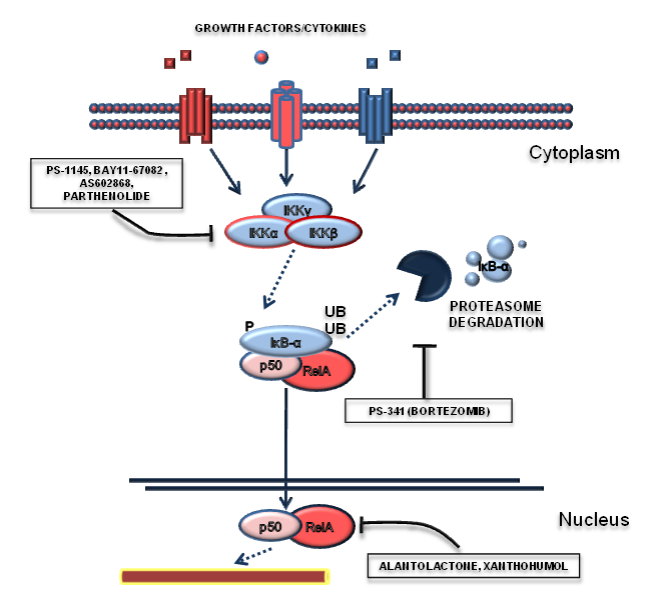
Targeting the NF-κB pathway This Figure reports those drugs that have been shown to modulate NF-κB in Ph^+^ leukemias. In particular, NF-κB signaling can be modulated by IKK inhibitors (such as PS1145, BAY11-67082, AS602868), proteasome inhibitors (Bortezomid) or with drugs able to act at the transcriptional level (alantolactone, parthenolide).

The important role of NF-κB in tumor cells and through the influence of the microenvironment suggests that this pathway can be therapeutically exploited for the treatment of Ph^+^ leukemias. Several small molecules inhibitors have been developed to target NF-κB-sustained cancers [84] and many of them have been tested in the context of Ph^+^ leukemias (Figure 4). The first observations of the efficacy of NF-κB targeting strategies come from CML and Ph^+^ ALL cellular models and were obtained with BAY11-67082, an inhibitor of the IκB-α phosphorylation [66], and Bortezomib (PS-341), which blocks proteasome-mediated IκB-α degradation [85-87]. Notably, the potential usage of Bortezomib in NF-κB-dependent contexts should have high priority in the design of early clinical trials due to its availability for in-human use and previous acquired clinical experience. NF-κB inhibitors appear promising also in combination with other agents, in particular in combination with tyrosine kinase inhibitors. IκB-α degradation inhibition with Bortezomib synergizes with the pro-apoptotic effects of Imatinib, when the two drugs are administered sequentially [88], suggesting that this strategy could be used in those patients who exhibit a partial response to TKI. It was also shown that the IKK inhibitors PS-1145 [72] and AS602868 [89] are able to promote CML primary cells apoptosis. In particular AS602868 presents an increased efficacy in BCR-ABL-T315I mutated cells [89]. In this specific cases, only Ponatinib is active against mutated Bcr-Abl kinase, although with a significant risk of major side effects [90]. Therefore, IKK inhibitors could be effective for the treatment of this specific mutation. Other compounds able to selectively target and inhibit NF-κB pathway have been described to be effective in Ph^+^ leukemias. In particular, Parthenolide has been shown to promote apoptosis of CML blast crisis cells [91], while Resveratrol [92], Xanthohumol [93], Curcumin [94], Alantolactone [95] were shown to be effective in other CML cell lines. Lastly, it is known that NF-κB is able to promote resistance to chemotherapeutic agents. Conventional chemotherapy is still necessary for the treatment of Ph^+^ ALL and CML blast crisis, due to reduced efficacy of TKI in these clinical scenarios. In line with these considerations, it was demonstrated that NF-κB inhibition restores sensitivity to Etoposide, a chemotherapeutic agent [92]. Overall, we can assert that NF-κB signaling may be therapeutically attackable, offering the chance to achieve synthetic lethality with TKIs and to respond -at least in part- to the unmet clinical need in the field of Ph^+^ leukemias.

## NEW TUMOR SUPPRESSIVE PERSPECTIVES: THE IκB-α/P53 CONNECTION

We have recently investigated the contribution of IκB-α in CML signaling [96]. IκB-α was generally described as a tumor suppressor and found deleted in about 20% of glioblastomas [97] and mutated in some cases of Hodgkin diseases [98]. In the attempt to observe IκB-α under-expression/abnormal localization in CML, we ended up to highlight a sustained IκB-α expression in CML primary samples [96]. Notably, we also observed that IκB-α is able to promote p53 nuclear exclusion in CML cells. IκB-α was previously reported to bind to both NF-κB and p53 [99-101]. Our data translated these observations in the setting of CML, describing a novel Bcr-Abl/IκB-α/p53 network, with potentially relevant implications for both tumorigenesis and therapy. In particular, Bcr-Abl/IκB-α complex promotes the nuclear exclusion and consequent inactivation of p53 [96], an essential tumor suppressor [102]. IκB-α can indeed be responsible for both the regulation of NF-κB and the triggering of p53 functional inactivation. Very recently, it was clearly demonstrated that CML CD34+ cells are characterized by the down-modulation of p53 targets and that drugs able to enhance p53 levels, together with c-MYC targeting drugs, promote CML cells selective apoptosis induction [103]. Indeed, the mechanisms of p53 modulation in CML pathogenesis appeared to be highly complex and, as a consequence, the development of strategies to reactivate p53 in CML and the effects of drugs that modulate IκB-α degradation (proteasome inhibitors) on p53 require further experimental investigations but may represent a new challenging approach to cure Ph+ leukemias.

## DISCUSSION

The NF-κB signal transduction is a very complex and stratified network, involving both canonical and non-canonical pathways and an integrated system of regulators. It plays an essential role in the pathogenesis of several cancers and offers several chances of therapeutic intervention with clinically available drugs. Almost 20 years ago, NF-κB was shown to play an essential role in the pathogenesis (and treatment) of Ph^+^ positive leukemias. While few controversial results have been reported in different cellular models and primary Ph^+^ samples, the following conclusions can be summarized. 1) NF-κB is aberrantly activated in Ph^+^ leukemias and in particular in CML blast phase and Ph^+^ ALL. The basal level of NF-kB activation in CML chronic phase is difficult to define accordingly to the methods of detection, samples management and appropriate controls. 2) Both Bcr-Abl signaling and non-cell autonomous regulation by cytokines, such as TNF-α and TGF-beta, cooperate to promote NF-κB activation in Ph^+^ leukemia. The existence of these two mechanisms of NF-κB activation may explain the differences observed in various Ph^+^ cell lines and primary cells: the removal of cells from the bone marrow environment could indeed affect the stromal contribution to NF-κB signaling. 3) In accordance to the previous point, murine modeling of NF-κB signaling in CML clearly demonstrates that IKK and IκB-α are mandatory to promote BCR-ABL tumorigenesis, ruling out the controversial results obtained in cell lines. 4) As observed in murine models, targeting IKK or preventing IkB-α degradation with proteasome inhibitors appear the best therapeutic interventions to inhibit NF-kB in Ph^+^ leukemias. Following the impressive results with the in vivo preclinical murine model, the association with TKI and IKK inhibitors should be tested with appropriate clinical trials to treat challenging Ph^+^ leukemias, such as CML blast crisis and Ph^+^ ALL. 5) Beside being a key NF-κB signaling inhibitor, IκB-α plays also an essential role in the regulation of p53 cellular compartmentalization and function, as we recently pointed out [96]. In particular, IκB-α acts at the crossroad between oncogenic signal (toward NF-κB) and tumor suppressive signal (toward p53). 6) The NF-IκB pathway may also be attractive for its ability to modulate p53 activity, even if further experimental approaches are mandatory to investigate this challenging therapeutic opportunity in the treatment of Ph+ leukemias.

As time has gone, during these 20 years the CML/NF-κB relationship has become more mature and has offered different chances to investigate the contribution of this pathway in tumorigenesis and cancer therapy.
